# The Physiology of Digestion, Considered with Relation to the Principles of Dietetics

**Published:** 1842-01

**Authors:** 


Art. IV.—*The Physiology of Digestion, considered with relation to the principles of Dietetics.* By Andrew Combe, m.d. Third Edition, revised and enlarged.—*Edinburgh,* 1841. 8vo, pp. 374.

Of this admirable work we have already more than once expressed our high opinion. It leaves nothing to be desired on the subject of which it treats, as far as regards the class of persons to whom it is principally addressed; and we are of opinion that there are few members of the profession who will not derive advantage from its perusal. In the present edition, among other improvements, we have a new chapter explaining more fully than heretofore the relation subsisting between the different kinds of food and the principal varieties of the human constitution.

